# Hydrogen Sulfide Ameliorates Homocysteine-Induced Cardiac Remodeling and Dysfunction

**DOI:** 10.3389/fphys.2019.00598

**Published:** 2019-05-24

**Authors:** Sumit Kar, Hamid R. Shahshahan, Tyler N. Kambis, Santosh K. Yadav, Zhen Li, David J. Lefer, Paras K. Mishra

**Affiliations:** ^1^ Department of Cellular and Integrative Physiology, University of Nebraska Medical Center, Omaha, NE, United States; ^2^ Department of Pharmacology and Experimental Therapeutics, Cardiovascular Center of Excellence, Louisiana State University Health Sciences Center, New Orleans, LA, United States; ^3^ Department of Anesthesiology, University of Nebraska Medical Center, Omaha, NE, United States

**Keywords:** hyperhomocysteinemia, H_2_S, fibrosis, hypertrophy, cardioprotection

## Abstract

Patients with diabetes, a methionine-rich meat diet, or certain genetic polymorphisms show elevated levels of homocysteine (Hcy), which is strongly associated with the development of cardiovascular disease including diabetic cardiomyopathy. However, reducing Hcy levels with folate shows no beneficial cardiac effects. We have previously shown that a hydrogen sulfide (H_2_S), a by-product of Hcy through transsulfuration by cystathionine beta synthase (CBS), donor mitigates Hcy-induced hypertrophy in cardiomyocytes. However, the *in vivo* cardiac effects of H_2_S in the context of hyperhomocysteinemia (HHcy) have not been studied. We tested the hypothesis that HHcy causes cardiac remodeling and dysfunction *in vivo*, which is ameliorated by H_2_S. Twelve-week-old male CBS^+/−^ (a model of HHcy) and sibling CBS^+/+^ (WT) mice were treated with SG1002 (a slow release H_2_S donor) diet for 4 months. The left ventricle of CBS^+/−^ mice showed increased expression of early remodeling signals c-Jun and c-Fos, increased interstitial collagen deposition, and increased cellular hypertrophy. Notably, SG1002 treatment slightly reduced c-Jun and c-Fos expression, decreased interstitial fibrosis, and reduced cellular hypertrophy. Pressure volume loop analyses in CBS^+/−^ mice revealed increased end systolic pressure with no change in stroke volume (SV) suggesting increased afterload, which was abolished by SG1002 treatment. Additionally, SG1002 treatment increased end-diastolic volume and SV in CBS^+/−^ mice, suggesting increased ventricular filling. These results demonstrate SG1002 treatment alleviates cardiac remodeling and afterload in HHcy mice. H_2_S may be cardioprotective in conditions where H_2_S is reduced and Hcy is elevated.

## Introduction

Elevated homocysteine in plasma and tissue (hyperhomocysteinemia, HHcy) increases the risk of developing cardiovascular disease (CVD). Mild HHcy (plasma homocysteine concentrations 15–30 μM) develops due to genetic deficiencies or polymorphisms in cystathionine-β-synthase (CBS) and methylenetetrahydrofolate reductase (MTHFR), folate and vitamin B12 deficiency, and a methionine-rich meat diet (Tyagi et al., 2005; Sen et al., 2010). Patients with diabetes also show HHcy possibly due to insufficient kidney function and the effects of glucose and insulin on decreased CBS activity and homocysteine tissue uptake (Fukagawa et al., 1986; Wollesen et al., 1999; Dicker-Brown et al., 2001; Ndrepepa et al., 2008; Ganguly and Alam, 2015). Mild HHcy is an independent risk factor for the development of cardiovascular disease and other pathologies including diabetes, hypertension, and birth defects (Hayden et al., 2004; Singh and Tyagi, 2017). Also, HHcy works synergistically with the detrimental effects of diabetes. Diabetic patients with HHcy are more likely to develop diabetic cardiomyopathy (DMCM) and are more than 10% more likely to die from CVD than diabetic patients without HHcy (Soinio et al., 2004; Tyagi et al., 2005). HHcy has also been associated with the development of DMCM in diabetic mouse models, and hyperglycemia-treated cardiomyocytes (Mishra et al., 2010a). However, while the detrimental effects of HHcy on CVD are well-established, few studies have investigated the molecular changes that HHcy induces on the heart.

Several placebo-controlled clinical trials have attempted to supplement folate, which metabolizes homocysteine to methionine, in order to reduce the detrimental effects of HHcy on CVD (Stampfer et al., 2006; Miller et al., 2010). Although all of these trials were able to reduce Hcy concentrations in serum, a meta-analysis showed no change in the relative risk of developing CVD (Bazzano et al., 2006). Also, folate treatment in diabetic mice with HHcy did not alleviate the development of DMCM (Mishra et al., 2010a). These negative findings suggest that the detrimental effects of HHcy are due to downstream products of Hcy metabolism and not Hcy itself.

Homocysteine is metabolized to cysteine by cystathionine-β synthase (CBS), and cystathionine γ-lyase (CSE) termed transsulfuration (Sen et al., 2010). Subsequently, the metabolism of cysteine by CBS and CSE produces hydrogen sulfide (H₂S). Hydrogen sulfide (H₂S) is an endogenous antioxidant gaseous signaling molecule that has been shown to have significant beneficial cardiovascular effects unlike homocysteine (Altaany et al., 2014; Polhemus et al., 2015). H₂S reduces hypertension (Sen et al., 2010; Sun et al., 2015) and has a cardioprotective effect in models of cardiomyopathy (Zhou et al., 2015; Liang et al., 2017).

Our lab has previously shown that HHcy mediates pathological cardiac remodeling, which is mediated in part by differential expressions of microRNA and altered beta-adrenergic signaling (Mishra et al., 2010a; Nandi and Mishra, 2017). In cultured cardiomyocytes, the administration of H₂S to HHcy-subjected cardiomyocytes mitigated the molecular and histological signs of hypertrophy and remodeling (Kesherwani et al., 2017). However, the effects of H₂S *in vivo* during HHcy are unknown.

We aimed to determine the molecular and histological changes in the heart induced by mild HHcy using CBS^+/**−**^ mice, which have been previously shown to have circulating levels of homocysteine approximately two times normal levels (Gupta et al., 2009; Azad et al., 2018). We further tested whether H₂S supplementation in CBS^+/**−**^ mice can mitigate cardiac remodeling *in vivo*.

## Materials and Methods

### Animals and H_2_S Donor Diet

**Figure 1:**
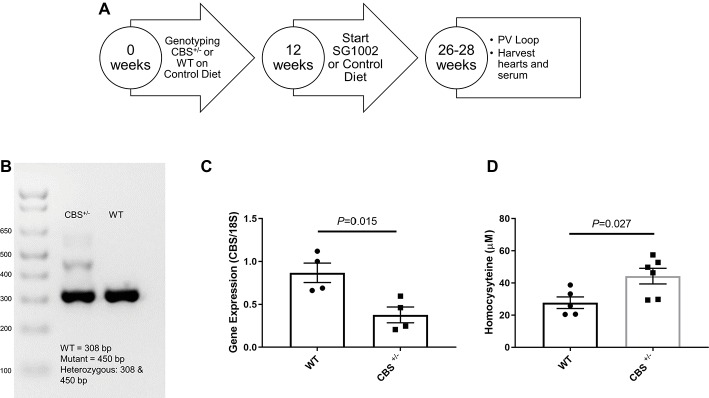
Experimental design and validation of HHcy in CBS^+/−^ mice. **(A)** Schematic of dietary schedule and cardiac function measurement. Mice were given an SG1002 (H_2_S donor) supplemented or control diet at 12 weeks of age for 14–16 weeks. **(B)** Representative genotyping of CBS^+/−^ and WT mice. **(C)** Gene expression of CBS measured by real-time PCR shows reduced transcript levels in the CBS^+/−^ heart at the end of the experiment compared to WT. **(D)** Homocysteine concentrations in serum were increased in CBS^+/−^ mice compared to WT mice confirming CBS^+/−^ mice are a model of hyperhomocysteinemia. Values expressed as mean ± SEM with dots representing each animal. Unpaired student t-test was used for statistical analysis.

We procured CBS^+/−^ mice from The Jackson Laboratory and bred these mice in the animal facility of the University of Nebraska Medical Center to obtain CBS^+/+^ (WT) mice. All animal studies were performed following the guidelines of the National Institutes of Health and the protocol approved by the *Institutional Animal Care and Use Committee* (*IACUC*) of the University of Nebraska Medical Center. The experimental design for the mice is shown in Figure 1A.

SG1002 is a proprietary slow release and orally active H_2_S donor (Sulfagenix, Inc). We started 12-week-old male CBS^+/−^ mice and their WT littermates on an SG1002 or control diet (307 mg of SG1002/kg of feed; Research Diets, Inc.; New Brunswick, NJ). The SG1002 diet achieved a dose of 40 mg/kg/d and was chosen based on previous clinical and preclinical studies of SG1002, which demonstrated stable increase in H_2_S from this dose (Kondo et al., 2013; Polhemus et al., 2015). Both diets contained a standard 15% kcal from fat. All groups were supplied with food and water ad libitum. Mice were selected randomly for the different treatment groups.

For genotyping, genomic DNA was extracted from ear punch tissue and was amplified using the protocol provided by The Jackson Laboratory. The forward primer sequence for CBS^+/+^ and CBS^+/−^ was 5′GATTGCTTGCCTCCCTACTG3′. The reverse primer sequence for CBS^+/+^ was 5′AGCCAACTTAGCCCTTACCC3′, and for CBS^+/−^ was 5′CGTGCAATCCATCTTGTTCA3′. cDNA was amplified in a BioRad cycler, and the PCR product was separated on a 2% agarose gel. WT mice had one band at 308 bp whereas CBS^+/−^ were characterized by two bands at 308 and 450 bp (Figure 1B).

### Hemodynamic Pressure-Volume Loop Recording

We measured hemodynamic changes in the heart by pressure-volume (PV) loop study following the best practice guidelines for invasive hemodynamic measurement in mice described by Lindsey et al. (2018). Mice were first anesthetized with 1–2% isoflurane, intubated and connected to a ventilator (MidiVent, Harvard Apparatus, Cambridge, MA). After opening the chest, a hole was made in the apex area of the left ventricle (LV) using a 27-gauge needle to insert a 4mm 1.2F catheter probe (Transonic, Ithaca, NY). The probe was advanced slowly into the LV so that it was away from the ventricle and septal walls as observed by the phase measurements of the surrounding muscle derived from the catheter. We maintained body temperature at 37°C by a temperature controller (ATC2000, World Precision Instruments, Sarasota, FL). Because heart rate can affect hemodynamics, we kept the heart rate between 450 and 500 beats per minute during PV recording by adjusting anesthesia. Volume measurements were calibrated automatically by the probe using admittance by removing parallel conductance from overall conductance and converted to an actual volume using the Weis equation accounting for body weight (Wei et al. 2005). The calculated stroke volume was verified in one WT animal by echocardiography. Hemodynamic parameters of diastolic and systolic function were determined by averaging at least 20 cardiac cycles from a baseline scan after proper probe placement was confirmed using the LabChart Pro PV Loop Analysis Add-On.

After recording was completed, mice were euthanized, organs were harvested, and cryopreserved at −80°C for later analyses. Approximately 400 μl of blood was collected from the inferior vena cava and after clot formation centrifuged 10 min at 2000× *g*, and the resulting serum was stored at −80°C in a microcentrifuge tube.

### H_2_S Measurement

H_2_S levels were measured in serum using gas chromatography, as previously described (Predmore et al., 2012). 0.2 ml blood, previously frozen at −80°C, was sealed with 1 M sodium citrate buffer (pH 6.0) and incubated at 37°C for 10 min with shaking at 125 rpm to release H_2_S gas. Then, 0.1 ml of the headspace gas was injected into a gas chromatograph (7890A GC System, Agilent, Santa Clara, CA) equipped with a dual plasma controller and chemiluminescence sulfur detector (355, Agilent) for H_2_S quantification against a standard curve. The samples were blinded for this measurement.

### Cysteine and Homocysteine Measurement

Cysteine concentrations in serum were measured by a fluorometric enzymatic assay (211099, Abcam) following the manufacturer’s protocol. A standard curve with five standards from 0 to 10 nM of cysteine was prepared in the manufacturer’s assay buffer. About 10 μl of neat serum was added to each well and incubated with enzyme mix and detection reagent. Homocysteine concentrations in serum were similarly measured by a fluorometric enzymatic assay (228559, Abcam). About 10 μl of neat serum was added to each well and incubated with enzyme mix and detection reagent. Each serum sample for the homocysteine assay was also run without enzyme mix in separate wells to measure background fluorescence.

The assay plate was read using a fluorescence plate reader (Glomax Multi+, Promega, Madison, WI) at an excitation wavelength of 365 nm and emission at 450 nm (cysteine assay) or at an excitation wavelength of 625 nm and emission at 708 nm (homocysteine assay). Readings were taken in endpoint mode 30 min after addition of the detection reagent and the fluorescence was used to calculate sample concentration against the standard curve plotted using a linear regression. Standards and samples were all run in duplicate and calculated concentrations were averaged from each well to determine sample concentration. Assay performance was verified by running quality control samples alongside samples which were within 10% of the nominal concentration.

### RNA Isolation and Quantitative PCR

RNA was isolated from the left ventricle using the mirVana isolation kit (AM1560, Thermo Fisher) which isolates both total and small RNA. RNA was quantified and verified for quality (260/280 > 1.8) using a NanoDrop One (Thermo Fisher). We used 1 μg of total RNA, and iScript cDNA synthesis (Bio-Rad) to synthesize cDNA, which was then amplified using SYBR green in a CFX qPCR instrument (Bio-Rad). We used 18S rRNA as an endogenous control. The primer sequences were: 18S (NR_003278), forward 5′GTAGTTCCGACCATAAACGA3′ and reverse 5′TCAATCTGTCAATCCTGTCC3′, and CBS (NM_144855) forward 5′TGCGGAACTACATGTCCAAG3′ and reverse- 5′TTGCAGACTTCGTCTGATGG3′. The melting temperature for all reactions was 55°C. Results were analyzed by CFX Manager 3.0 software (Bio-Rad).

### Western Blotting

Protein was extracted from frozen portions of the LV by using RIPA buffer (BP-115D, Boston BioProducts, Ashland, MA) and a 2 ml glass homogenizer. Protein lysates were quantified using a BCA protein assay kit (23227, Thermo Fisher). About 30 μg of proteins loaded onto 10 or 12% SDS PAGE gels.

All membranes were blocked using 5% nonfat dry milk in Tris-buffered saline (TBS) at room temperature for 30 min. The primary antibodies used were: β-MHC (1:1,000, ab172967, Abcam), CSE (1:1500, H00001491-M02, Abnova), c-Fos (1:1,000, 2250 s, Cell Signaling), total c-Jun (1:1,000, 9165 s, Cell Signaling), MMP9 (1:1,000, ab119906, Abcam), and TGF-β1 (1:100, sc146, Santa Cruz) for 1 h. Secondary antibody staining was performed using: anti-mouse IgG-HRP, and anti-rabbit IgG-HRP (used at half the concentration of the respective primary antibody, Cell Signaling). Restore Stripping Buffer (46430, Thermo Fisher) was used as necessary. Protein expression was normalized against total protein measured by Ponceau staining. Bands were visualized with a ChemiDoc Imaging System (Bio-Rad), and band intensity was analyzed by ImageLab software (Bio-Rad).

### Histological Analysis

Tissue sections of mouse hearts were made for visualizing cell size and collagen fibers. We made 5 µm transverse sections of hearts embedded in paraffin. Sections were fixed in 10% formalin. Staining was performed by the UNMC Tissue Sciences Facility using Sirius red for collagen staining and H&E staining following standard protocols. We also performed wheat germ agglutinin (WGA) staining on 5 μm cryosections of the heart. Sections were fixed in 4% paraformaldehyde and incubated in 5 μg/ml of WGA (W834, Thermo Fisher) for 10 min at room temperature.

Sections were imaged using a VWR bright field microscope and Motic Images Plus 2 imaging tool (Motic, Richmond, British Columbia). WGA sections were observed under a fluorescence microscope (EVOS, Life Technologies). We calculated perivascular and interstitial (INT) fibrosis by quantifying percentage red pixels/total pixels in a uniform area using Image J software. Exposure settings for imaging and thresholds for red pixel detection were kept consistent for all images. Two random areas of the LV were chosen and the same areas were imaged in all hearts. Fibrosis percentage was averaged across the two areas to determine interstitial fibrosis for each animal. For perivascular fibrosis, a similar diameter vessel was identified in all sections and two random areas around the vessel were quantified for percentage fibrosis and averaged. Hypertrophy was quantified by manually counting the number of cells in a uniform area of the image. Cells were counted in two randomly chosen areas of LV section, which were kept consistent in all hearts, and averaged for each animal.

### *In situ* Zymography

MMP activity was visualized in tissue sections by *in situ* zymography. 5 μm transverse sections of the heart were made in a cryostat. Sections were incubated with fluorescein-conjugated DQ gelatin (D12054, Thermo Fisher), which yields highly fluorescent peptides when digested by MMPs, for 1.5 h at room temperature in the dark. After washing in phosphate buffered saline, sections were fixed with 4% paraformaldehyde, stained with DAPI for 5 min and mounted with a coverslip. Some sections were treated with 1 mM 1,10-phenanthroline monohydrate, an inhibitor of all MMPs, as a negative control.

### Statistical Analysis

All statistical analyses were performed using GraphPad Prism 8 software. One-way ANOVA and Tukey’s multiple comparisons were used to determine the statistical variance between the four groups. Unpaired student t-test was used for statistical analysis between two groups. *p* < 0.05 was considered statistically significant and individual *p* values are shown for all graphs.

## Results

### Animals and Gravimetric

We confirmed CBS^+/−^ mice by genotyping shortly after birth and by reduced gene expression of CBS measured by qPCR of left ventricle tissue at the end of the experiment (Figure 1). Serum concentrations of homocysteine in CBS^+/−^ mice were approximately twice the concentrations seen in WT mice confirming the presence of mild HHcy in CBS^+/−^ mice (Figure 1D). At 12 weeks of age, WT control and CBS^+/−^ mice were given a normal chow control diet or a diet with orally active SG1002. The diet continued for 14–16 weeks (Figure 1A) at which time the chest was opened to measure cardiac function, organs and serum were removed, and wet organ weight was measured (Table 1). Heart weight was significantly increased in CBS^+/−^ mice which were normalized in CBS^+/−^ mice that were given the SG1002 diet. Body weight and weight of other organs were unchanged between groups.

**Table 1:** Gravimetric Data from WT and CBS^**+/−**^ mice with and without SG1002 diet.

Parameter	WT	CBS^+/−^	CBS^+/−^ + SG1002	WT + SG1002
Age (Days)	195 ± 3.90	192 ± 2.43	193 ± 8.06	199 ± 11.3
Body weight (g)	24.5 ± 0.67	25.7 ± 1.36	26.7 ± 1.20	26.6 ± 0.72
Heart weight (mg)	115 ± 3.77	129 ± 6.40*	116 ± 4.87^#^	124 ± 3.35
Liver weight (mg)	1,030 ± 102	973 ± 57.0	881 ± 44.3	978 ± 38.8
Lung weight (mg)	133 ± 4.06	126 ± 4.30	126 ± 3.37	133 ± 1.89
Kidney weight (mg)	148 ± 5.68	160 ± 4.59	139 ± 4.69^#^	144 ± 3.81

* *p* < 0.05 vs. WT;

# *p* < 0.05 vs. CBS+/−.

### HHcy and H_2_S Alters Transsulfuration Pathway

**Figure 2:**
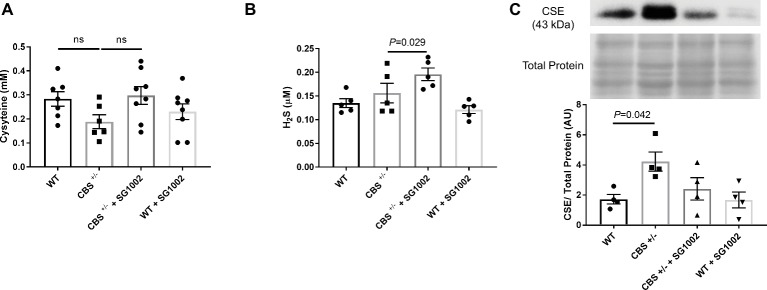
HHcy and SG1002 alter the transsulfuration pathway. **(A)** Serum concentration (mM) of cysteine in CBS^+/−^ and SG1002 treated mice at the end of the experiment. No statistically significant difference was observed between groups. **(B)** Serum H_2_S did not decrease in CBS^+/−^ mice, but it was elevated by SG1002 diet. **(C)** Representative and quantified Western blot analysis of CSE. Total protein from ponceau staining was used as a loading control. The H_2_S producing enzyme CSE increases in CBS^+/−^ mice. All values expressed as mean ± SEM with dots representing each animal. One-way ANOVA and Tukey’s post- hoc test were used for statistical analysis.

To determine if HHcy in CBS^+/−^ mice and SG1002 H_2_S donor diet induces changes in the transsulfuration pathway, we measured serum concentrations of cysteine and H_2_S and expression of CSE in the heart (Figure 2). Cysteine, which is produced by CBS from homocysteine, showed slightly, but not statistically significant, decreased concentrations in CBS^+/−^ mice compared to WT mice. H_2_S concentrations were also measured in serum at the end of the experiment. H_2_S concentrations were not reduced in CBS^+/−^ mice; however, the SG1002 diet in these mice did significantly increase H_2_S concentrations (Figure 2B). Protein expression of the H_2_S producing enzyme CSE was significantly increased in the left ventricle of CBS^+/−^ mice.

### Improved Cardiac Fibrosis With H_2_S in HHcy

**Figure 3:**
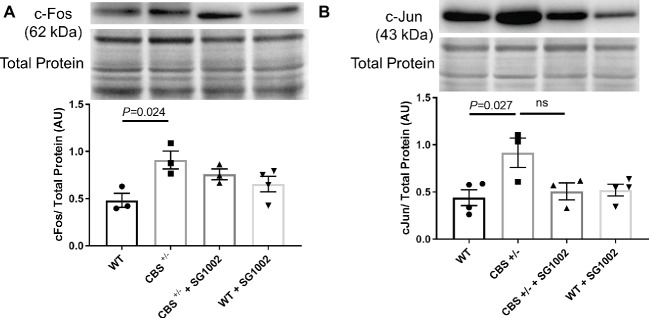
Signals of early cardiac remodeling are elevated in HHcy. **(A)** Representative and quantified Western blot analysis of c-Fos. Total protein from ponceau staining was used as a loading control. c-Fos expression increases in the CBS^+/−^ heart. **(B)** Representative and quantified Western blot analysis of c-Jun. Total protein from ponceau staining was used as a loading control. c-Jun expression increases in CBS^+/−^ mice. All values expressed as mean ± SEM with dots representing each animal. One-way ANOVA and Tukey’s post- hoc test were used for statistical analysis.

To investigate whether CBS^+/−^ mice develop cardiac fibrosis, we measured early markers of remodeling and histological and molecular markers of fibrosis in the left ventricle. C-Fos and c-Jun protein expression in the left ventricle were significantly elevated in CBS^+/−^ mice compared to WT mice (Figure 3). SG1002 administration did not significantly reduce this expression although there was a trend for decreased c-Jun expression compared to CBS^+/−^ mice.

**Figure 4:**
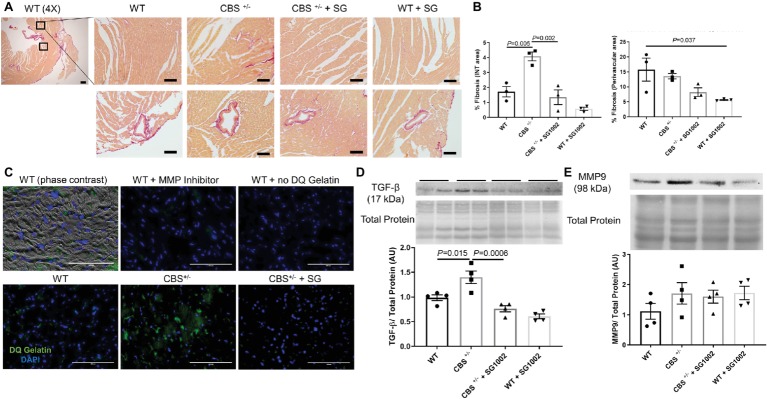
Increased fibrotic remodeling in the CBS^+/−^ heart is improved by H_2_S. **(A)** Representative sirius red collagen staining in LV. The top panel shows interstitial area and bottom panel shows perivascular fibrosis at 4× and 40× stage and 10x eyepiece magnifications. The same two areas of the LV were assessed and averaged for interstitial fibrosis in all groups. Scale bar = 200 μm. **(B)** Image J quantification of sirius red collagen staining in the interstitial area (left) and perivascular area (right). Interstitial fibrosis increased significantly in CBS^+/−^ mice which was reversed with SG1002 dietary treatment. Perivascular fibrosis is reduced in WT mice with SG1002. **(C)** *In situ* zymography of left ventricular cryosections. Green staining is DQ gelatin indicating MMP collagenase/gelatinase activity. Blue staining is DAPI nuclei stain. Scale bar = 100 μm. DQ staining overlaid with phase contrast imaging of tissue suggests DQ staining occurs in the extracellular matrix. Use of 1,10-phenanthroline general MMP inhibitor nearly eliminated DQ staining. CBS^+/−^ mice showed increased DQ staining indicating increased MMP activity which was reduced by SG1002 treatment. **(D)** Representative and quantified Western blot analysis of TGF-β showed increased expression in CBS^+/−^ mice which was reduced with SG1002 treatment. Total protein from ponceau staining was used as a loading control. **(E)** Representative and quantified Western blot analysis of MMP9 which was unaltered in all groups. Total protein from ponceau staining was used as a loading control. All values expressed as mean ± SEM with dots representing each animal. One-way ANOVA and Tukey’s post- hoc test were used for statistical analysis.

We found increased collagen deposition in the left ventricle of CBS^+/−^ mice by picosirius red, which stains all types of collagen indicating increased cardiac fibrosis (Figure 4A). This increase was limited to the interstitial myocardium and not the perivascular area (Figure 4B).

*In situ* zymography, an assay for matrix metalloprotease (MMP) activity, was qualitatively increased in the left ventricle of CBS^+/−^ mice compared to WT activity (Figure 4C). Overlay of phase contrast images with DQ gelatin stain from sections demonstrated that DQ staining occurring mostly in the extracellular space and application of the MMP inhibitor 1,10-phenanthroline abolished all DQ signal confirming the specificity of the DQ staining for MMP collagenase/gelatinase fibrotic activity.

Finally, molecular markers and pro-fibrotic mediators were measured. TGF-β protein expression was increased in the CBS^+/−^ left ventricle (Figure 4D). MMP9 expression (Figure 4E), however, was not increased indicating increased collagen deposition is likely due to an increase in MMP activity rather than expression.

H_2_S donor administration by diet mitigated the histological and molecular signs of cardiac fibrosis. Interstitial fibrosis normalized to WT levels in CBS^+/−^ mice. MMP activity also decreased in CBS^+/−^ + SG1002 mice to levels similar to WT. Finally, TGF-β protein expression was restored to WT levels in CBS^+/−^ mice treated with SG1002.

### Improved Cardiac Hypertrophy With H_2_S in HHcy

**Figure 5:**
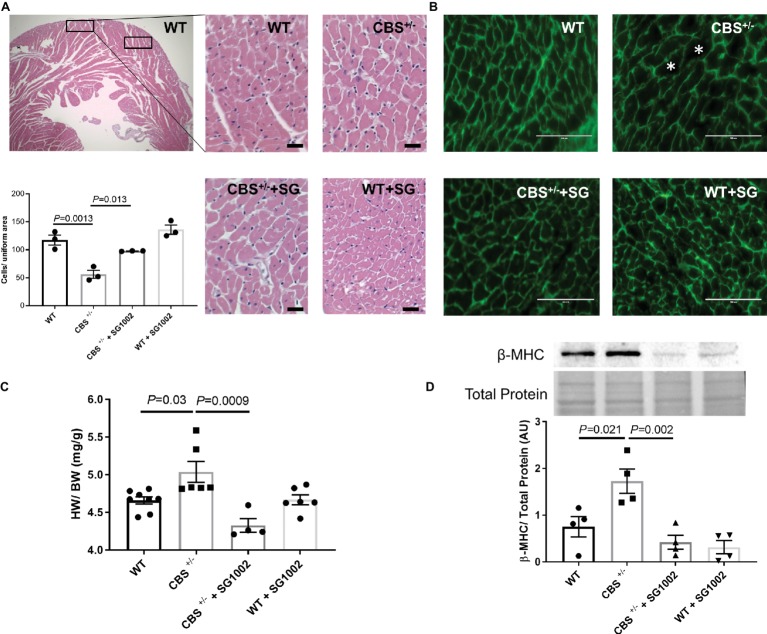
Increased hypertrophic remodeling in the CBS^+/−^ heart is improved by H_2_S. **(A)** Representative and quantification of cardiomyocyte size by H&E staining. CBS^+/−^ mice showed cardiomyocyte hypertrophy (cells/uniform area), which was normalized by SG1002 treatment. Scale bar = 30 μm. **(B)** Representative WGA staining of left ventricle heart sections. Green color represents cardiomyocyte boundary and “*” denotes a hypertrophied cardiomyocyte. Scale bar = 100 μm. **(C)** SG1002 diet reduced heart weight/body weight (HW/BW) in CBS^+/−^ mice. **(D)** β-MHC, a marker of hypertrophy, was increased in CBS^+/−^ mice but was restored by SG1002 treatment. Total protein from ponceau staining was used as a loading control. All values expressed as mean ± SEM with dots representing each animal. One-way ANOVA and Tukey’s post- hoc test were used for statistical analysis.

We subsequently measured histological and molecular markers of cardiac hypertrophy in CBS^+/−^ mice with and without SG1002 diet. First, we measured cardiomyocyte size in H&E and WGA stained sections of the left ventricle (Figures 5A,B). CBS^+/−^ mice sections showed fewer cells per uniform area measured indicating greater cell size and cardiac hypertrophy. Similarly, heart weight to body weight ratio was increased in CBS^+/−^ compared to WT hearts (Figure 5C). Finally, fetal/neonatal beta-cardiac myosin heavy chain (β-MHC) is commonly re-expressed in the adult heart during cardiac hypertrophy and remodeling. CBS^+/−^ mice hearts showed increased protein expression of β-MHC providing further confirmation of cardiac hypertrophy in HHcy mice (Figure 5D).

H_2_S donor diet normalized all signs of cardiac hypertrophy in CBS^+/−^ mice. Cell size was reduced in H&E stained sections, heart weight to body weight ratio was reduced, and β-MHC protein expression was reduced to levels comparable to WT hearts.

### Alterations in Hemodynamic Function With H_2_S

**Figure 6:**
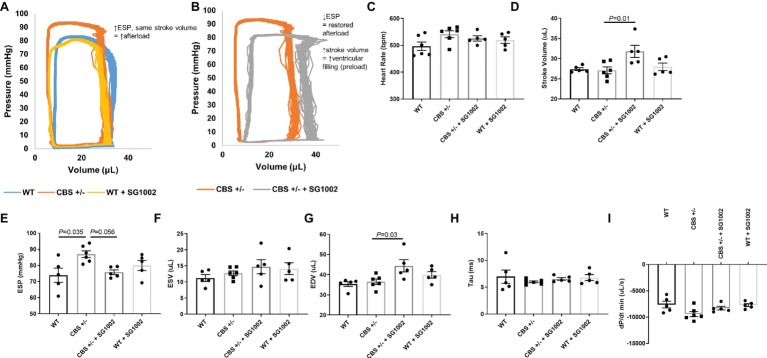
SG1002 treatment ameliorates increased afterload in CBS^+/−^ mice. Representative PV loop measurement in **(A)** Representative PV trace in WT, CBS^+/−^, and WT mice treated with SG1002. **(B)** Representative PV trace in CBS^+/−^ mice treated with or without SG1002. **(C)** Heart Rate. **(D)** Stroke volume. **(E)** End-systolic pressure. **(F)** End-systolic volume. **(G)** End-diastolic volume. **(H)** and **(I)** Measures of diastolic function (Tau and dP/dt min) were unchanged between groups. One-way ANOVA and Tukey’s post-hoc test were used for statistical analysis.

Cardiac function and hemodynamics were evaluated at the end of the experiment using PV loop recordings to determine if the cellular remodeling alterations in HHcy mice resulted in altered cardiac function (Figure 6). We evaluated measures of systolic and diastolic function as cardiac remodeling affects diastolic function first (Swynghedauw, 1999). CBS^+/−^ mice exhibited increased end systolic pressure compared to WT mice but no change in end-diastolic or systolic volumes. Thus, stroke volume and cardiac output remained similar to WT mice. This depicts a scenario of increased afterload but no decrease in systolic function. SG1002 restored the increased afterload in CBS^+/−^ mice by reducing end systolic pressure. Also, end-diastolic volume was significantly increased in CBS^+/−^ + SG1002 mice (Figure 6G) while end-systolic volume was unchanged. This resulted in increased stroke volume in the CBS^+/−^ + SG1002 group and suggested an increase in preload and ventricular filling.

Measures of diastolic function such as Tau and dp/dt min were unchanged between all groups. Load independent measures of cardiac function such as the end-diastolic PV relationship were not evaluated in this study.

## Discussion

**Figure 7:**
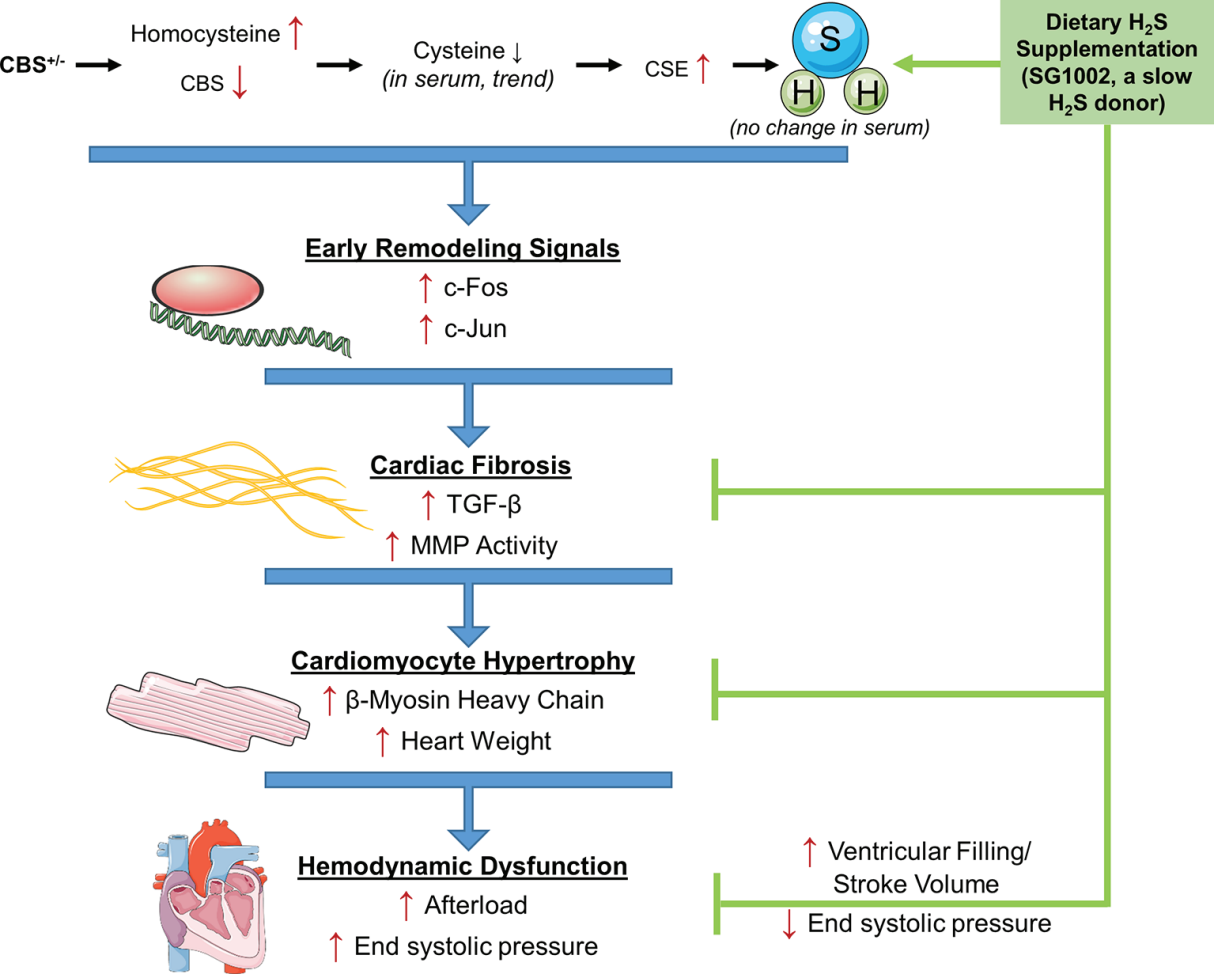
Working model for the amelioration of cardiac remodeling in HHcy by H_2_S. During pathological remodeling, transcriptional changes lead to inflammation and cell death. Fibrosis formation and hypertrophy of cardiomyocytes follow to fill the growing vacant extracellular space and lead to cardiac dysfunction. CBS^+/−^ HHcy mice showed alterations in the transsulfuration pathway and the molecular and histological signs of remodeling, fibrosis, and hypertrophy. H_2_S supplementation with SG1002 inhibited these alterations in pathological remodeling and reduced afterload in the heart.

While it is well established that HHcy increases the risk of CVD, the molecular mechanisms of the effects of Hcy on the heart are less clear. Also, H_2_S, a by-product of Hcy transsulfuration, may reverse these detrimental effects where folate has not. We investigated the effects of HHcy on the mouse heart in the CBS^+/−^ model of HHcy and tested whether H_2_S can reverse these effects *in vivo*. Our results show that CBS^+/−^ mice demonstrated multiple signs of early and late cardiac remodeling including fibrosis and hypertrophy which culminated in increased afterload on the heart (Figure 7). Remodeling changes were inhibited by dietary administration of the SG1002 H_2_S donor to the mice for ~14 weeks and increased cardiac stroke volume. Our data further suggest H_2_S reduces TGF-β signaling *in vivo* to protect against cardiac fibrosis and hypertrophy.

Several studies have investigated the molecular effects of HHcy on vascular function, but few have studied cardiac remodeling and cardiac function (Lentz et al., 2000; Wang et al., 2003; Sen et al., 2012). The present study suggests that HHcy increases the risk of CVD in part because of cardiac remodeling, specifically increased cardiac fibrosis and hypertrophy. Our results are in agreement with a study by Raaf et al., which observed increased collagen deposition in the myocardium of HHcy rats (Raaf et al., 2011). This pathology also correlated with an increase in expression of the transcription factors and cytokines c-Fos, c-Jun, and TGF-β in HHcy mice. The master pro-fibrotic cytokine TGF-β uses c-Fos and c-Jun in its signaling cascade to mediate TGF-β-induced transcription of genes that increase collagen deposition in the heart (Zhang et al., 1998; Khalil et al., 2017). It has also been shown that the TGF-β/c-Jun/c-Fos pathway alters MMP expression and activity (Matrisian, 1990). Our study and the study by Raaf et al. showed similar increases in TGF-β expression and MMP activity in the heart during HHcy.

Many types of cardiovascular diseases which involve cardiac remodeling such as DMCM also involve a cascade of inflammation, oxidative stress, cell death which results in fibrosis and compensatory cardiomyocyte hypertrophy (Lorenzo et al., 2013; Fuentes-Antras et al., 2015). It is possible in HHcy that the cardiac remodeling we observed is because of inflammation, cell death, and oxidative stress on the heart rather than an effect of TGF-β signaling alone. In support of this hypothesis, other studies of HHcy in the heart and liver demonstrate increased oxidative stress and increased pro-apoptotic Bax protein expression (Robert et al., 2005; Chang et al., 2008).

Previous studies have not however determined if remodeling manifests in alterations in cardiac function. In our study, CBS^+/−^ mice did not show drastic deterioration of cardiovascular function, aside from an increase in afterload (increased end systolic pressure with conserved stroke volume) on the heart and did not show functional signs of cardiomyopathy even though they showed abnormal cardiac fibrosis and hypertrophy. Studies have shown alterations in vascular function and more notably hypertension in HHcy (Sen et al., 2010). Hcy has been shown to cause endothelial injury directly, promote atherosclerotic lesion formation, and reduce nitric oxide (NO) availability, all of which can induce hypertension (Wang et al., 2003; Sen et al., 2010). The increased afterload on the heart may be due to these arterial changes. It is also likely that an additional hit is necessary in addition to HHcy to induce more drastic functional cardiovascular alterations such as decreased diastolic function. For example, it appears that diabetes acts synergistically with HHcy to accelerate the development of cardiomyopathy (Mishra et al., 2010b). Other techniques such as load independent PV loop measurements or cardiac MRI may be more sensitive to detect early alterations in cardiac function.

We hypothesized that H_2_S, a metabolite of Hcy, could mitigate the effects of HHcy *in vivo*. H_2_S is cardioprotective against fibrosis and hypertrophy in models of HHcy, ischemia/reperfusion, and diabetic cardiomyopathy (Barr et al., 2015; Xie et al., 2015; Nandi and Mishra, 2017); however, *in vivo* validation of these data is limited. We found that increasing circulating concentrations of H_2_S by dietary administration of a slow release H_2_S donor, SG1002, normalized the molecular and histological measurements of fibrosis and hypertrophy. H_2_S significantly reduced collagen deposition in the left ventricle of HHcy mice, reduced MMP activity, reduced cardiomyocyte size, and heart weight normalized to body weight. H_2_S did not normalize c-Fos and c-Jun expression, however, did significantly reduce TGF-β expression. Thus, the cardioprotective effects of H_2_S in HHcy may still be due to its effects on TGF-β signaling. In rat models of hypertension, H_2_S donors similarly reduced collagen remodeling in the heart which was associated with effects on TGF-β signaling (Sun et al., 2014; Meng et al., 2015). The cardioprotective benefits of H_2_S may also be due to its role in signaling of inflammation, oxidative stress, cell death, and master transcription factors (Guo et al., 2013; Sun et al., 2015; Zhou et al., 2015; Kar et al., 2019). For instance, an *in vitro* study by Yang et al. demonstrated in fibroblasts that H_2_S sulfhydrated Keap1 to activate the Nrf2 transcription factor, production of the antioxidant glutathione, and protected cells for early death (Yang et al., 2013). Further studies are necessary to determine the mechanisms of H_2_S-mediated cardioprotection in HHcy.

Functionally, increasing H_2_S normalized the increased afterload observed in HHcy mice and increased stroke volume suggesting an increase in preload. While it is unclear why preload and ventricular filling would be enhanced in this group, we hypothesize that these effects may also be due to vascular changes from the systemic H_2_S donor. H_2_S has been shown to reduce vascular smooth muscle cell proliferation (Yang et al., 2004), increase NO production in vascular smooth muscle cells (Jeong et al., 2006), and inhibit angiotensin-converting enzyme (ACE) activity of endothelial cells (Laggner et al., 2007).

It is important to note that we expected H_2_S and cysteine levels to decrease in HHcy CBS^+/−^ mice since CBS deficient mice cannot transsulfurate Hcy into H_2_S. In fact, in the rat liver and heart in HHcy, H_2_S levels were decreased due to decreased CSE activity (Yao, 1975; Li et al., 2009). In our study, however, H_2_S and cysteine levels in serum were unchanged in HHcy. We attribute this to the compensatory increase in tissue level CSE expression which is consistent with our previous *in vitro* data (Nandi and Mishra, 2017). Thus, in our study CBS deficient mice developed cardiac remodeling, presumably by altered Hcy levels itself or possibly a local change in H_2_S concentrations that were not measured in this study. Elevating H_2_S in HHcy prevents these effects which suggest a clinical benefit of H_2_S in HHcy where trials of administering folate in HHcy have provided no benefit.

In conclusion, we demonstrate that H_2_S prevents cardiac remodeling induced by HHcy *in vivo* and induces molecular alterations in TGF-β expression and MMP activity. These results suggest that H_2_S may be a preventative treatment for cardiac remodeling and in patients with HHcy and in conditions where Hcy is elevated such as in diabetic patients with DMCM.

## Data Availability

All datasets generated for this study are included in the manuscript and/or the supplementary files.

## Ethics Statement

This study was carried out in accordance with the recommendations of The University of Nebraska Medical Center, IACUC committee.

## Author Contributions

HS, SK, TK, SY, and ZL contributed to data generation. PM conceived the idea. SK and PM contributed in drafting the manuscript. DL provided SG1002 compound, and all the authors reviewed and approved the manuscript.

### Conflict of Interest Statement

The authors declare that the research was conducted in the absence of any commercial or financial relationships that could be construed as a potential conflict of interest.
